# Dysregulated dendritic cells in sepsis: functional impairment and regulated cell death

**DOI:** 10.1186/s11658-024-00602-9

**Published:** 2024-05-30

**Authors:** Li-yu Zheng, Yu Duan, Peng-yi He, Meng-yao Wu, Shu-ting Wei, Xiao-hui Du, Ren-qi Yao, Yong-ming Yao

**Affiliations:** 1 Translational Medicine Research Center, Medical Innovation Research Division of the Chinese PLA General Hospital, 28 Fuxing Road, Haidian District, Beijing, 100853 China; 2 Department of Critical Care Medicine, Affiliated Chenzhou Hospital (the First People’s Hospital of Chenzhou), Southern Medical University, Chenzhou, 423000 China; 3 Department of General Surgery, The First Medical Center of Chinese PLA General Hospital, 28 Fuxing Road, Haidian District, Beijing, 100853 China

**Keywords:** Sepsis, Dendritic cells, Functional impairment, Regulated cell death, Immunomodulation

## Abstract

Sepsis is defined as life-threatening organ dysfunction caused by a dysregulated host response to infection. Studies have indicated that immune dysfunction plays a central role in the pathogenesis of sepsis. Dendritic cells (DCs) play a crucial role in the emergence of immune dysfunction in sepsis. The major manifestations of DCs in the septic state are abnormal functions and depletion in numbers, which are linked to higher mortality and vulnerability to secondary infections in sepsis. Apoptosis is the most widely studied pathway of number reduction in DCs. In the past few years, there has been a surge in studies focusing on regulated cell death (RCD). This emerging field encompasses various forms of cell death, such as necroptosis, pyroptosis, ferroptosis, and autophagy-dependent cell death (ADCD). Regulation of DC’s RCD can serve as a possible therapeutic focus for the treatment of sepsis. Throughout time, numerous tactics have been devised and effectively implemented to improve abnormal immune response during sepsis progression, including modifying the functions of DCs and inhibiting DC cell death. In this review, we provide an overview of the functional impairment and RCD of DCs in septic states. Also, we highlight recent advances in targeting DCs to regulate host immune response following septic challenge.

**Graphical Abstract:**

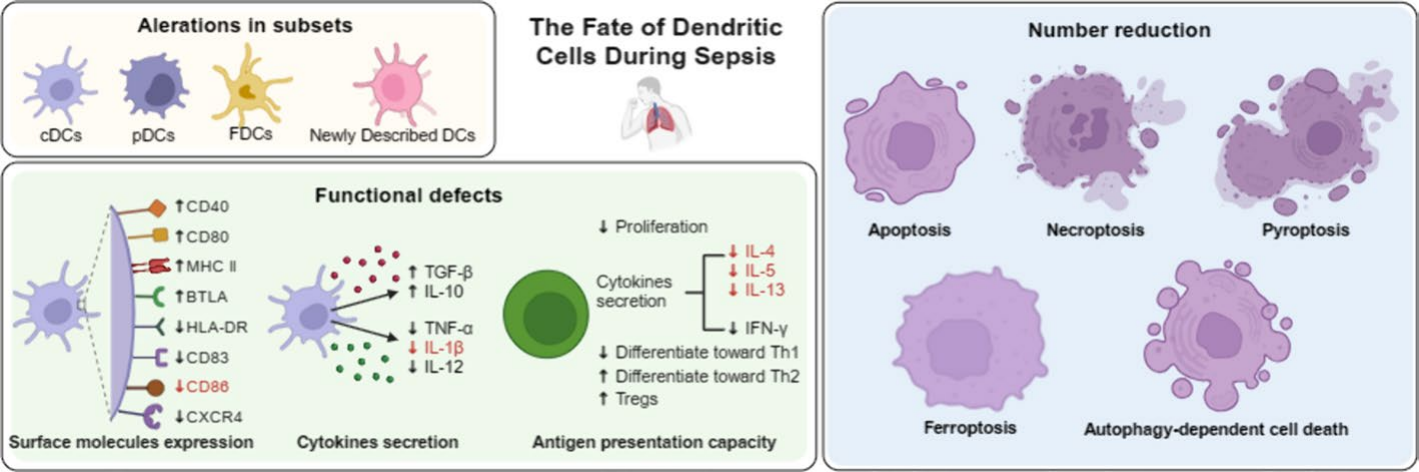

## Introduction

According to the Third International Consensus Definition for Sepsis and Septic Shock (Sepsis 3.0), sepsis is defined as life-threatening organ dysfunction caused by dysregulated host responses to infection [1]. Sepsis is a major complication in patients admitted to the medical intensive care unit (ICU) and has long been recognized as the primary factor contributing to mortality in critically ill patients [2]. It possesses the characteristics of high morbidity and mortality, along with frequent incidence of sequelae [3]. In line with the recently published epidemiological data, there are roughly 48.9 million new sepsis cases, with more than 11 million deaths in the world annually [4]. In-hospital mortality of septic patients has declined over the past decades, attributed to earlier recognition of sepsis and best-practices supportive therapies [5]. The pathophysiological mechanisms underlying sepsis appear to be complicated, including imbalance of inflammatory response, immunosuppression, coagulation disorders, etc. [3]. A comprehensive of the pathogenesis of sepsis holds significant theoretical importance and practical value in terms of its clinical diagnosis, treatment, and prognosis [6]. Recent findings suggest that immune dysfunction is a crucial factor in the progression of sepsis, as the majority of septic individuals have encountered instances of lymphopenia. However, the specific mechanisms that cause sepsis-induced immunosuppression at the cellular and molecular levels still need to be elucidated [7, 8]. To aid in our understanding of the pathophysiological process of sepsis, it is significantly helpful to comprehend the alterations in different immune cell subsets in the setting of sepsis. This knowledge can potentially provide a therapeutic target for immune-modulatory strategies [9]. The aim of this review is to investigate the potential involvement of dendritic cells (DCs) in sepsis, taking into account their significant impact on the host's immune response.

DCs serve as proficient antigen-presenting cells (APCs), connecting innate immunity with adaptive immunity. They possess the ability to identify harmful microorganisms, display antigens, trigger adaptive immunity, and promote the development of autoimmune immune tolerance. The involvement of DCs in the development of immune dysregulation after sepsis onset is widely recognized [10]. Specifically, DCs exhibit abnormal functions and obviously decreased numbers in sepsis [11–13]. DCs can be divided into several subsets based on location, ontogeny, and functions [14]. It is worth mentioning that recent studies have discovered new categories of DCs through the use of advanced techniques such as single-cell RNA sequencing (scRNA-seq) and cytometry by time-of-flight (CytoF), which enable high-throughput analysis [15–17]. Studies have suggested that previously neglected DC populations may be necessary for certain immunopathologies [18, 19]. Several studies have consistently reported that the decreases in DCs counts are closely related to the elevated mortality rates and incidences of nosocomial infection among patients with sepsis [20–23]. Notably, a substantial reduction in DCs can be primarily attributed to initiating a cell death program caused by sepsis. It was found that DC reduction was mainly mediated by caspase-3-dependent apoptotic pathways, whereas newly published studies revealed that the other forms of programmed cell death (PCD) could contribute to the depletion of DCs in sepsis. Given the pathophysiological significance of the reduction of DCs in pathogenesis and development of sepsis, herein we summarized the recent advances in the PCD of DCs during sepsis, including apoptosis, necroptosis, pyroptosis, ferroptosis, and autophagy-dependent cell death (ADCD). Recent studies have shown that novel immunomodulatory interventions that target DCs can reduce morbidity and mortality in sepsis and septic shock by modifying the immune functions of DCs and inhibiting DC cell death [13, 22, 24–26]. Considering the crucial function of DCs in the onset and advancement of sepsis, this review aims to consolidate the research advancements on DCs in sepsis. It particularly focuses on the emerging forms of PCD in DCs during septic exposure, aiming to enhance comprehension of the immune pathogenesis of sepsis and consequently offer new targets for immunomodulation.

## Classification of DC subsets

**Fig. 1:**
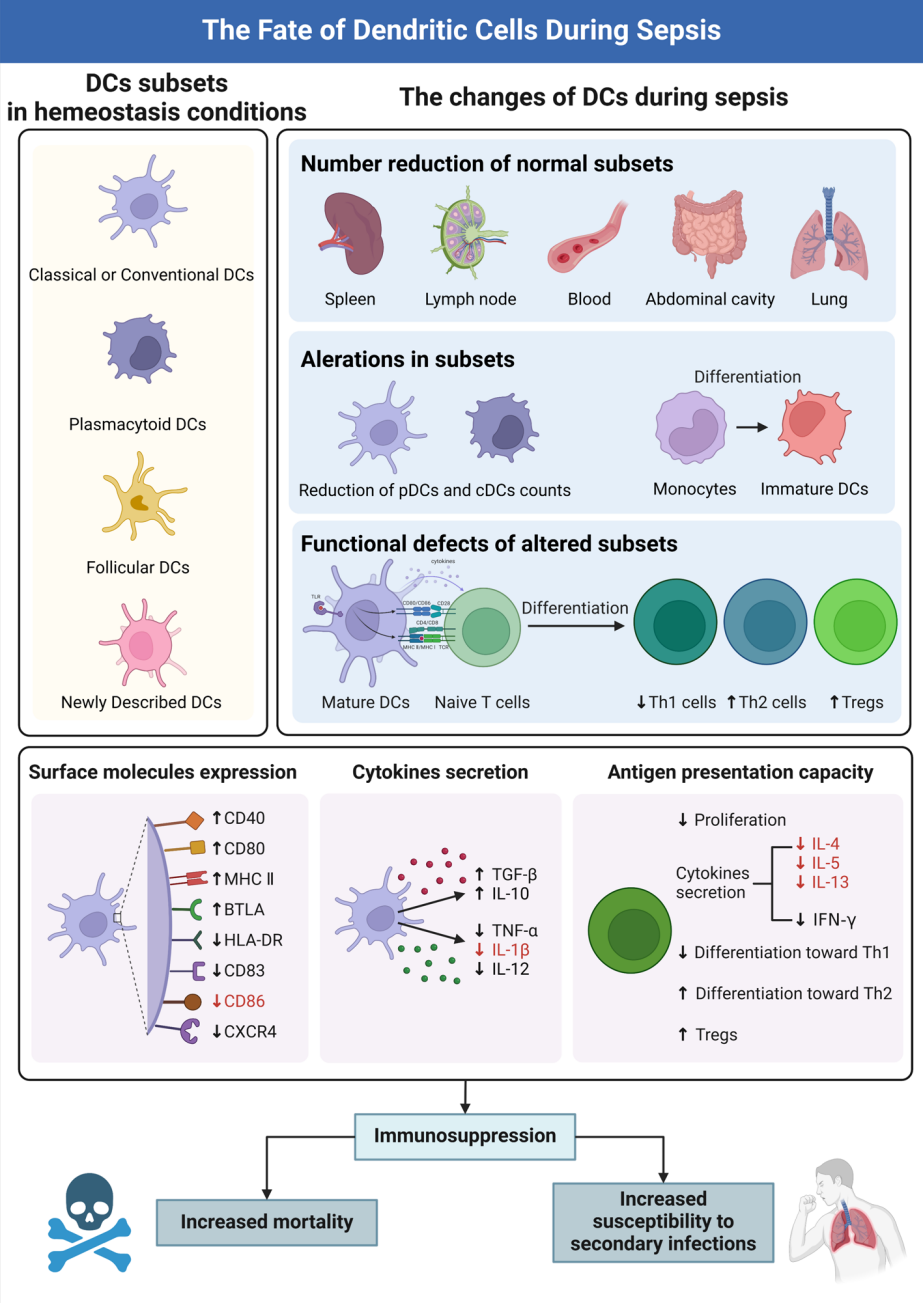
The fate of dendritic cells (DCs) during sepsis. The upper left panel shows the different subsets of DCs in homeostasis conditions. The upper right panel shows the changes in DCs during sepsis, including number reduction of normal subsets, alteration in subsets, and functional defects of altered subsets. The lower panel shows three aspects of DC dysfunction in a septic state, including the expression of surface molecules, cytokine secretion, and antigen presentation capacity. These changes will lead to the formation of an immunosuppressive environment, which is closely associated with increased mortality and susceptibility to secondary infections in patients with sepsis. DCs, dendritic cells; cDCs, classical or conventional DCs; pDCs, plasmacytoid dendritic cells; FDCs, follicular dendritic cells; MHC, major histocompatibility complex; MHC-II, MHC class II; HLA-DR, human leukocyte antigen-DR; IL, interleukin; IFN, interferon; TNF, tumor necrosis factor; TGF, transforming growth factor; Tregs, regulatory T cells; Th, T helper cells

DCs are a class of bone-marrow-originated cells differentiating from lymph-myeloid hematopoietic stem cells. Their developmental trajectory depends on the synergetic effect of transcription factors that facilitate lymph-myeloid differentiation [27, 28]. Since each DC subset exerts a unique function, it is critical to understand the physiological characteristics across disparate subsets of DCs [29]. DCs can be roughly categorized into classical or conventional DCs (cDCs), plasmacytoid DCs (pDCs), and follicular dendritic cells (FDCs) [27, 30–33] (Fig. 1). cDCs originating from common myeloid progenitors (CMPs) in bone marrow express high levels of CD11c and the major histocompatibility complex (MHC)-II, making them the most potent APCs in vivo. Corresponding to different phenotypic markers and functions, they can be further divided into two subsets: cDC1 and cDC2. Phenotypically, cDC1s are characterized as high expression of CD141, whereas upregulated CD1c is noted in cDC2s [29]. At the functional level, cDC1s represent critical players in anti-virus and anti-tumor immunity through cross-presenting intracellular antigens to cytotoxic CD8^+^ T cells (CTLs) via MHC-I [32]. In contrast, cDC2s possess a potent intrinsic capacity to present extracellular antigens, parasites, and allergens to helper CD4^+^ T cells (Th) through MHC-II expression [32, 34]. Langerhans cells (LCs) are a specific type of cDC that mainly gathers in peripheral non-lymphoid tissues. These cDCs have a low level of MHC-II and costimulatory molecules, but they have a high level of Toll-like receptors (TLRs), modulatory receptors, and chemokine receptors. Their ability to absorb and process antigens is potent, whereas the capacity to present antigens remains relatively weak [35]. pDCs are identified as CD11c^dim^ CD123^+^, derived from bone marrow common lymphoid progenitors (CLPs) [29]. pDCs have a round plasma cell-like morphology and express intermediate levels of MHC-II and costimulatory molecules, enabling pDCs to present antigens to CD4^+^ T cells. However, they typically express a group of TLRs such as TLR7 and TLR9, which mainly recognize microbial dsRNA, ssDNA, or bacterial/viral CpG DNA, rendering its key role in preventing virus infection. Upon activation, it has the ability to release a significant quantity of type I interferon (IFN-α/β) [32]. FDCs are developed from mesenchymal progenitor cells (MPCs), distributed in lymph nodes, spleen, lymph follicles, and germinal centers of the mucosal immune system [36]. FDCs lack the ability to present antigens owing to the absence of MHC-II and costimulatory molecules. FDCs can effectively capture antigen–antibody complex, antigen-complement complex, and antigen–antibody-complement complex through highly expressed IgG Fc receptor and C3b/C3d receptor [36–39]. FDCs attract B cells by producing and releasing CXC chemokine ligand (CXCL)13. The B cells then efficiently recognize, ingest, and process the antigen or immune complex on the FDCs’ surface [40, 41]. A group of mature DCs with an abundance of immunoregulatory molecules, referred to as “mature DCs enriched in immunoregulatory molecules” (mregDCs), was recently discovered in non-small cell lung cancer through scRNA-seq [19]. In our previous investigation, we examined the diversity of immune cell subsets in a murine sepsis model using scRNA-seq. We observed a significant increase in spleen-resident mregDCs shortly after the cecal ligation and puncture (CLP) procedure, indicating their involvement in the hyperinflammatory phase of sepsis. Furthermore, the existence of mregDCs in bronchoalveolar lavage fluid (BALF) of individuals with sepsis was substantiated by utilizing up-to-date findings from a single-cell investigation of subjects with COVID-19 [18].

## The functional impairment of DCs in sepsis

The crucial involvement of DCs in the onset and progression of septic complications has been widely acknowledged, as both the function of DCs and the total number of DCs undergo significant alterations during sepsis. Many studies mainly focus on the functions of DCs, of which three essential aspects can be characterized (Fig. 1).

### Surface molecular expression

Mature DCs become dysfunctional upon sustainable septic insults [46]. Functional markers such as CD40, CD80, CD86, and MHC-II were significantly upregulated by DCs in animal sepsis models during the initial phase of sepsis, which is essential for the activation of T cells, while DCs downregulated the expression of surface molecules at the late stage of sepsis [42]. Furthermore, clinical findings indicated that the presence of HLA-DR on DCs was notably reduced in individuals suffering from sepsis, indicating its crucial prognostic significance for patients experiencing immune suppression [12, 43].

### Cytokine secretion

The cytokines secreted by DCs are obviously altered during sepsis [12, 44–46]. There is a proposal suggesting that the secretion pattern of cytokines in DCs is atypical in sepsis, leading to a significant decrease in the release of proinflammatory cytokines [tumor necrosis factor (TNF)-α, IL-1β, and IL-12]. On the other hand, there is a significant increase in the production of anti-inflammatory cytokines (such as TGF-β and IL-10), showing similar characteristics to the endotoxin tolerance observed in monocytes [12, 42, 45, 47]. Human DCs treated with IL-10 exhibit suppressor activity specific to antigens, thereby contributing to the development of anergic T cells [48]. TGF-β promotes the accumulation of regulatory T cells (Tregs) in lung-induced immune paralysis and forms an immunosuppressive environment in sepsis [23].

### T cell-stimulatory capacity

It has been demonstrated that the ability of DCs to stimulate T cells is significantly diminished in cases of sepsis [49]. The proof of this can be seen in the decrease in T cell growth, the lower production of cytokines like IL-2, and the higher IFN-γ/IL-4 ratio, suggesting a change in T cell polarization toward the Th2 pathway [50]. The production of DCs by hematopoietic stem cells and hematopoietic progenitor cells (HSPCs) is hindered by systemic inflammation caused by sepsis [51]. Furthermore, the makeup of DC subcategories experienced notable modifications [12]. The quantities of cDCs and pDCs in the bloodstream of septic individuals decreased significantly, while the transformation of monocytes into CD1a^−^ DCs intensified, consequently inducing T cell anergy and fostering Treg proliferation [52–54]. Furthermore, DCs can manifest as an immature state upon septic challenge, which produce large amounts of IL-10 instead of IL-12, inducing an anergic profile of T cells and a propensity toward Tregs [42, 54].

In the setting of sepsis, the mechanisms with regard to impaired functions of DCs are largely unknown. Possible reasons for this could be linked to the following aspects. Firstly, the endoplasmic reticulum stress (ERS) serves as an internal self-defense mechanism. Moderate ERS is conducive to restoring cell homeostasis under external stimulation; prolonged or excessive ERS impairs ER function, resulting in autophagy and/or apoptosis. At the early stage of sepsis, the activation of ERS facilitates the maturation and activation of DCs and promotes T cell proliferation and polarization toward Th1. At the late stage of sepsis, an overabundance of ERS may lead to apoptosis of DCs [55]. Secondly, newly discovered negative immunoregulatory proteins, such as tumor necrosis factor α-induced protein 8 like-1 (TNFAIP8L2, TIPE1) and TIPE2 from tumor necrosis factor α-induced protein 8 family, have been found to inhibit the maturation and activation of DCs in septic mice. Studies indicate that TIPE1 inhibits the maturation of DCs and subsequent T-cell-mediated immunity via the programmed cell death-ligand 1 (PD-L1)/programmed death 1 (PD-1) signaling pathway [56]. TIPE2 inhibits DC immune function by suppressing autophagy through the TGF-β-activated kinase 1 (TAK1)/c-Jun N-terminal kinases (JNK) pathway [57]. Thirdly, organelle-specific autophagy, which is a significant subtype of autophagy, specifically aims to degrade various organelles in order to maintain their quality. In sepsis, the dysfunction of DCs was prevented by regulating the quality control of mitochondria through protein tyrosine phosphatase (PTEN)-induced putative kinase 1 (PINK1)-mediated mitophagy, as indicated by a report [58].

## Regulated cell death of DCs in sepsis

Of note, another significant alteration is the decrease in the amount of DCs upon septic challenge [24, 59–62]. Studies on animal models of sepsis and human sepsis have found a marked depletion of DCs in lymphoid and non-lymphoid organs [9, 63–65]. In a study using CLP mice, they noticed that the splenic CD11c^+^ DCs underwent evident apoptosis through the caspase-3 pathway at 12–36 h after the onset of sepsis, thereby resulting in significantly decreased DC number in the abdominal cavity [59, 66–69]. The reduction of DCs was directly related to the prognosis and the incidence of nosocomial infection in patients [20, 22, 23]. Hence, keeping track of the quantity of DCs can offer an initial valuable evaluation of the seriousness concerning the disruption of the host’s immune response to infection, which could aid in forecasting fatal consequences in patients with sepsis and offer a fresh approach for treating sepsis-induced immunosuppression [13, 22].

For the past 10 years, the Nomenclature Committee on Cell Death (NCCD) has consistently revised the categorization of cell death on the basis of morphological, biochemical, and functional viewpoints [70]. Cell death is now classified into accidental cell death (ACD) and RCD on the basis of functional status. ACD means the instantaneous and catastrophic demise of cells exposed to severe physical, chemical, or mechanical insults. In stark opposition to ACD, RCD depends on specialized molecular apparatus, suggesting that it can be influenced (i.e., postponed or expedited) through pharmacological or genetic interventions. ACD is an uncontrolled biological process, while RCD consists of well-organized signaling cascades and specific molecular effector mechanisms. RCD under physiological conditions is also referred to as PCD. Currently known types of RCD can be divided into several subtypes in terms of molecular basis, including apoptosis, necroptosis, pyroptosis, ferroptosis, ADCD, and so on.

### Apoptosis

Apoptosis is the term used to describe genetically determined processes that selectively remove unnecessary, permanently impaired, or potentially dangerous cells [71]. Apoptotic death of immune cells has been extensively studied in sepsis, and it plays a crucial role in immune hyporesponsiveness and even organ dysfunction [72]. As a bridge linking innate immunity with adaptive immunity, apoptosis of DCs appears to be critically involved in immunosuppression secondary to septic insults [44, 72, 73].

Numerous researchers have identified two primary routes of apoptosis: the intrinsic pathway, also known as the mitochondrial pathway, and the extrinsic pathway, alternatively referred to as the death receptor pathway. Intrinsic or mitochondrial pathways can be triggered by stimuli mediated oxidative stress, mitochondrial disorder, and DNA damage, including anti-tumor agents, hypoxia, ischemia–reperfusion injury, and ionizing radiation. Damage to the mitochondria results in increased permeability of the outer membrane of the mitochondria, causing a significant release of cytochrome *c* into the cytoplasm. This cytochrome *c* then binds with apoptotic protease activating factor-1 (APAF-1), initiating the cascade of apoptosis by activating pro-caspase 9 and forming a complex known as the “apoptotic body.” Ligands binding to death receptors, such as TNF–TNF receptor (TNFR)1, factor-associated suicide ligand (FasL)-factor-associated suicide (Fas), and TNF-related apoptosis-inducing ligand (TRAIL)–TRAIL receptor (TRAILR), induce extrinsic or death receptor pathways. The caspase protease family mediates the convergence of internal and external pathways, resulting in the development of characteristic apoptotic traits such as DNA fragmentation, chromatin condensation, cell shrinkage, and membrane blistering. In addition, it is noteworthy that the extrinsic pathway triggers intrinsic mitochondrial apoptosis by activating caspase-8. Both intrinsic and extrinsic pathways can be influenced by signaling cascades, including p53, nuclear factor κ-B (NF-κB), ubiquitin–proteasome system, and phosphoinositide 3 kinase (PI3K) pathways, indicating extensive crosstalk between these two apoptotic pathways.

**Fig. 2:**
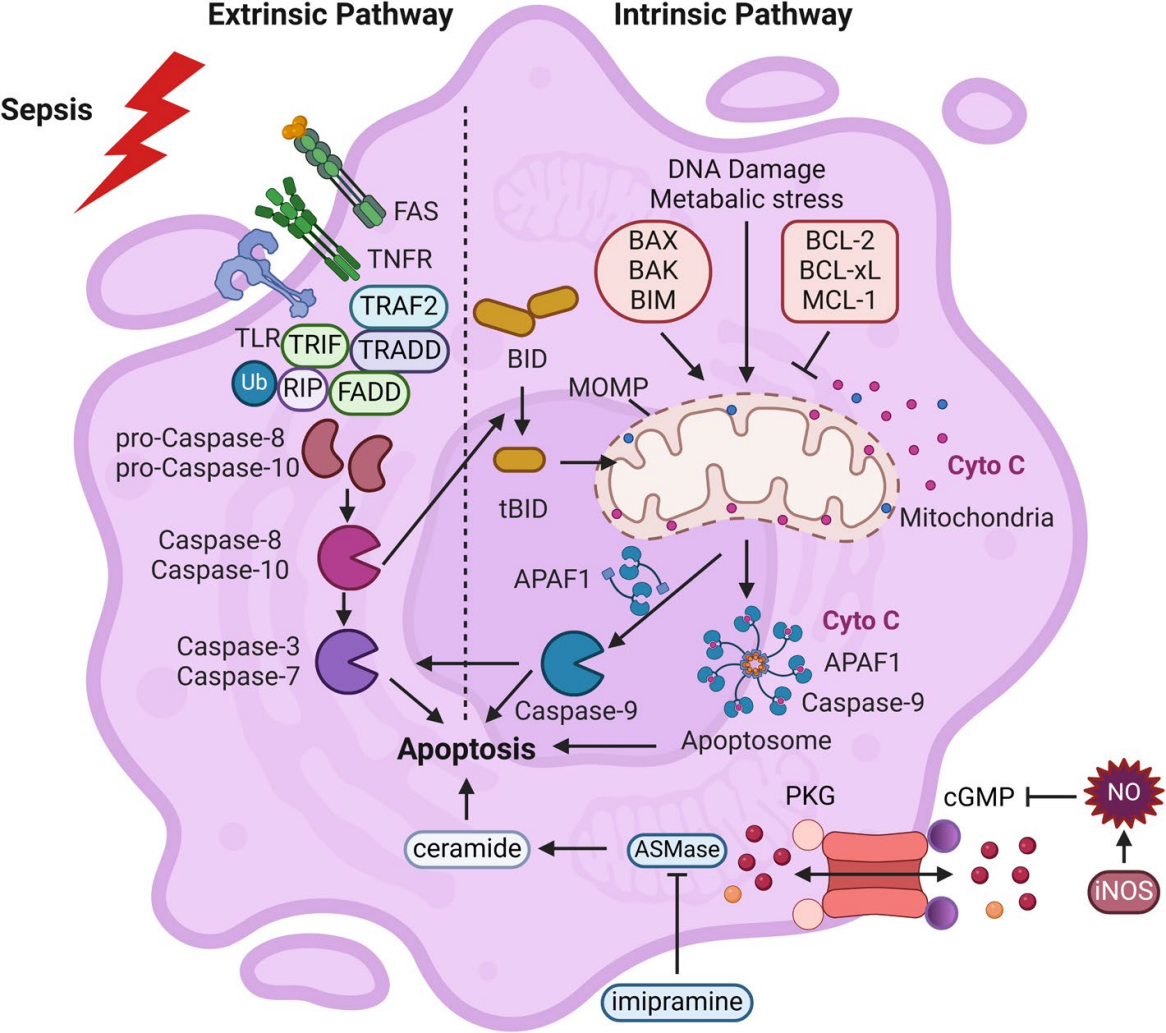
Apoptosis of dendritic cells (DCs) in sepsis. The apoptosis of DCs in sepsis mainly includes intrinsic or mitochondrial pathway and extrinsic or death receptor signaling. There is extensive crosstalk between these two apoptotic pathways; the extrinsic pathway triggers the intrinsic pathway by activating caspase-8 to produce truncated bid (tBid), and the intrinsic pathway can amplify the extrinsic pathway by activating caspase-3/-7 with activated caspase-9. The interaction of these two pathways eventually leads to typical apoptotic features, such as DNA fragmentation, chromatin condensation, cell shrinkage, and membrane blistering. Nonclassical apoptosis of DCs in sepsis is accompanied by the generation of ceramide through the activation of A-SMase. This pathway can be antagonized by NO and A-SMase inhibitors including imipramine. TLR, Toll-like receptor; TNFR, TNF receptor; FADD, Fas-associated with death domain protein; RIP, receptor-interacting protein; TRAF2, TNF receptor associated factor 2; TRADD, TNFRSF1A-associated via death domain; TRIF, Toll/interleukin-1 receptor domain containing adaptor inducing IFN-β; APAF-1, apoptotic protease activating factor-1; MOMP, mitochondrial membrane potential; A-SMase, acid sphingomyelinase; PKG, cGMP-dependent protein kinases; iNOS, inducible nitric oxide synthase

Since the apoptosis process of DCs is largely context dependent concerning different stimuli, we mainly discuss the mechanism underlying the apoptosis of DCs upon septic insults. It was observed in both human and murine sepsis models that caspase-3-mediated apoptosis of DCs led to the loss of DCs, resulting in immunosuppressive status and increased mortality [68, 73]. By employing a caspase-3 inhibitor or generating *Caspase-3*^−/−^ mice, along with the upregulation of the anti-apoptotic protein B-cell lymphoma-2 (Bcl-2) in DCs mice (referred to as *DCs-hBcl-2* mice), the survival of DCs was enhanced, the immunosuppression induced by lipopolysaccharide (LPS) was attenuated, and the resistance to lethal endotoxic shock was increased. Consequently, this led to an improvement in the unfavorable consequences of sepsis [66, 67, 74]. Further studies showed that pro-apoptotic and anti-apoptotic proteins were closely associated with the occurrence of apoptosis of DCs in sepsis. For example, mice with *Bim* knockout significantly decreased apoptosis of DCs during sepsis induction [75]. Other reports have indicated that sepsis-induced apoptosis of DCs accompanied with ceramide generation by activating acid sphingomyelinase (A-SMase). LPS and TNF-α induced proinflammatory response and apoptosis of DCs in mice were substantially mitigated by being treated with the A-SMase inhibitor including imipramine. Notably, A-SMase involvement in apoptosis was more common in immature DCs as immature DCs were more sensitive to ASMase-induced apoptosis. In a sepsis mouse model, the activation of cGMP-dependent protein kinases (PKG) by nitric oxide (NO) counteracted the apoptosis of immature DCs induced by A-SMase. In septic mice, the apoptosis of immature DCs was significantly increased when inducible nitric oxide synthase (*iNOS*^−/−^) was knocked out [76]. In addition, apoptosis of splenic DCs can be activated through TLR4 and TLR2 signaling pathways, followed by activating interferon regulatory factor 1 (IRF1) through a TLR4-dependent, myeloid differentiation primary response gene 88 (MyD88)-independent manner [59, 77]. Moreover, it has been documented that a pathway independent of TLR4 engagement can trigger LPS-induced apoptosis of DCs via CD14 and activate calcineurin-activated T nuclear factor (NFAT) [78]. So far, the mechanism concerning sepsis-induced apoptosis of DCs has not been fully elucidated. Further study of its specific mechanism will provide a new therapeutic strategy for managing sepsis-induced immunosuppression related to apoptosis of DCs (Fig. 2).

### Necroptosis

Necroptosis is mainly mediated by TNFR and TLR family members, IFN, intracellular RNA, and DNA sensors. Afterward, receptor proteins are interacted with by protein kinases such as receptor-interacting protein kinase (RIPK)1 and RIPK3, which transmit death signals and phosphorylate mixed-lineage kinase domain-like protein (MLKL). MLKL acts as an initiator of cell death and eventually induces necroptosis [79]. The process of necroptosis leads to the liberation of various molecular patterns associated with damage (DAMPs), such as mitochondrial DNA (mtDNA), high mobility group box-1 protein (HMGB1), and lactate dehydrogenase (LDH). These substances further enhance and intensify the inflammatory cascade, significantly worsening the clinical outcomes of sepsis patients [80, 81].

**Fig. 3:**
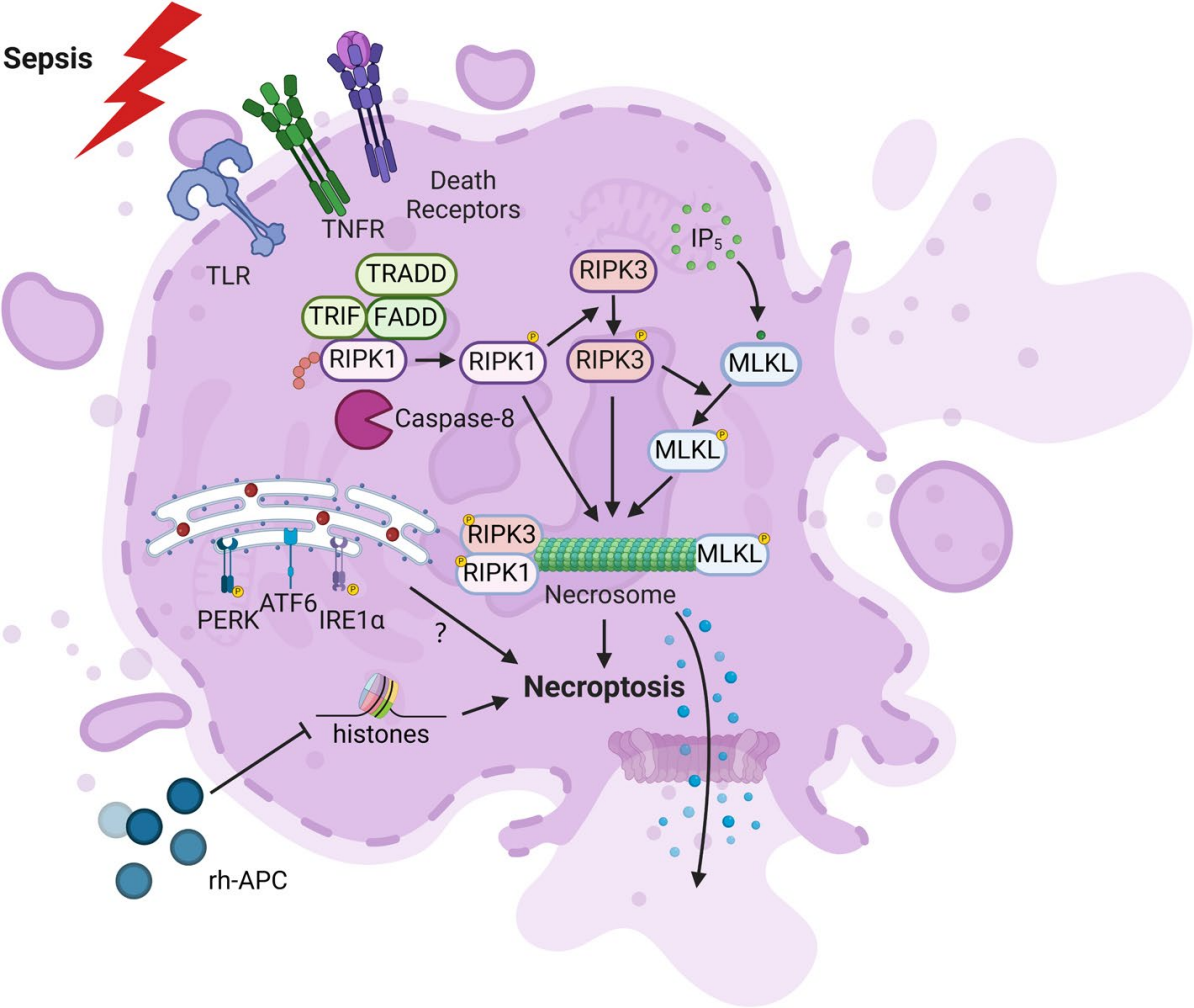
Necroptosis of dendritic cells (DCs) in sepsis. Activation of multiple cellular receptors can trigger necroptosis. These include death receptors (e.g., Fas), TLR, and TNFR. After the receptor is activated, it binds to the adaptor protein, resulting in the downstream recruitment of RIPK1, which is deubiquitinated and then phosphorylated, followed by phosphorylation of RIPK3 by p-RIPK1 and phosphorylation of MLKL by p-RIPK3. Taken together, the phosphorylated forms of these three form necrosomes, which punch holes in the cell membrane and subsequently lead to cell rupture and leakage of cell contents. In addition, histones can induce necroptosis, and rh-APC can rescue it. Also, ERS-related proteins are involved in the occurrence of necroptosis, but the detailed mechanism is still unrevealed. TLR, Toll-like receptor; TNFR, TNF receptor; FADD, Fas-associated with death domain protein; TRAF2, TNF receptor associated factor 2; TRADD, TNFRSF1A-associated via death domain; TRIF, Toll/interleukin-1 receptor domain containing adaptor inducing IFN-β; RIPK, receptor-interacting protein kinase; MLKL, mixed-lineage kinase domain-like protein; PERK, PKR-like endoplasmic reticulum kinase; ATF6, activating transcription factor 6; IRE1α, inositol-requiring enzyme 1α; rh-APC, recombinant human-activated protein C

Emerging evidence has suggested that the RIPK1–RIPK3–MLKL-mediated necroptosis and the release of large amounts of DAMPs can increase mortality in TNF-α-induced sepsis. *Ripk3*^−/−^ showed a marked protective effect on mice after CLP operation, thus significantly reducing the mortality of septic animals [82–84]. Additional research has indicated that necroptosis takes place concurrently in the liver, intestines, and lungs during sepsis, thereby playing a role in the emergence of multiple organ dysfunction syndrome (MODS) in sepsis [85–87]. In addition, the markers of necroptosis RIPK1, RIRK3, and MLKL and the HMGB1 released by necroptosis in peripheral blood of septic patients were significantly increased and positively correlated with the severity and mortality attributed to sepsis [88, 89]. Using necroptosis inhibitors such as Nec-1, GSK2982772, and ZB-R-55 could alleviate sepsis-induced acute liver and lung injury, release inflammatory mediators in serum, and reduce mortality in septic mice [90–94]. Further data revealed that downregulation of RIPK3 expression reduced necroptosis, which might be related to its ability to affect the transcription of activating transcription factor 6 (ATF6) and mitigate excessive ERS [92]. Research conducted on monocyte-derived dendritic cells (MDDCs) in the peripheral blood of individuals with septic shock revealed that MDDCs in patients who survived primarily experienced apoptosis through a caspase-dependent pathway, whereas MDDCs in patients who did not survive were exposed to the necroptotic pathway. Circulating histones are identified as critical mediators of DC necrotizing cell death, which could be rescued by recombinant human-activated protein C (rh-APC) [95]. Notably, other studies showed that the treatment with Nec-1 could not improve the survival of septic mice [96]. This paradoxical phenomenon may be attributed to differences in the dosages of Nec-1 and animal models [97, 98]. These contradictory results are not fully explained. Nevertheless, both of them confirmed that RIPK1 kinase activity is essential for the survival of animals subjected to sepsis [99]. As knockout *Ripk1* could result in death in mice, O’Donnell et al. constructed *Ripk1* ^DC KO^ mice, and the experiments demonstrated that necroptosis of DCs might underlie the hyperinflammatory syndrome and immunosuppression in severe sepsis [100]. It is important to conduct additional research to elucidate the exact regulatory pathway of necroptosis in DCs during sepsis (Fig. 3).

### Pyroptosis

Pyroptosis represents an RCD commonly initiated by inflammasomes, characterized by cell swelling, membrane blebbing, DNA fragmentation, and eventually cell lysis. The occurrence of pyroptosis relies on the inflammatory caspase and the Gasdermin protein family. The classical pyroptosis pathway is often described as occurring through a two-step process. NF-κB is activated to induce the expression of various proteins, assembling a complex called the inflammasome in the “activating signal” step. Typically, inflammasomes are composed of a cytosolic pattern recognition receptor [PRR; for example, members of the NOD-like receptor (NLR) family, NLRP1, NLRP3, and NLRC4], an adaptor protein containing the CARD domain (such as ASC), and pro-caspase-1. Of note, one well-accepted approach to monitor pyroptotic activity is to analyze inflammasome activation by detecting NLRP3 and visualizing ASC specks. Regarding the subsequent phase of activation, following the cleavage of Gasdermin D (GSDMD) by caspase-1, the N-terminal portion of GSDMD assembles into clusters and generates pores in the cell membrane, ultimately resulting in cell disruption and the liberation of cytokines. Concomitantly, pro-IL-1β and pro-IL-18 are activated by proteolysis to generate their active forms, which are secreted from the cell via the pores. Therefore, pyroptosis serves as a vital natural defense mechanism and significantly contributes to the body's ability to fend off harmful pathogens [101].

In recent decades, a growing number of studies have investigated pyroptosis and its relationship with sepsis [102–104]. Previously, pyroptosis was thought to occur only in monocytes or macrophages, while subsequent results indicated that it could also occur in other cell types [105]. Moderate pyroptosis is beneficial for the body to clear the pathogens, while excessive pyroptosis will lead to host immune dysfunction, multi-organ dysfunction, and even death [106–108]. In sepsis, the occurrence of macrophage pyroptosis was observed, and the survival rate of septic mice was enhanced by inhibiting the inflammatory response of macrophages through NLRP3 knockout, leading to an improvement [109]. It is likely that different ways to reduce lipid peroxidation markedly reduced the mortality of septic mice by decreasing pyroptosis of macrophages [110]. However, whether or not pyroptosis of DCs occurs in sepsis remains controversial. Erlich et al. noted that only monocytes and macrophages were involved in pyroptosis [111]. Guermonprez et al. reported that DCs had a class of iDCs manifested as M-CSFR^+^CD209a^+^ that could develop into pyroptosis [112]. In recent years, our team confirmed that pyroptosis of CD11c^+^CD11b^int^MHC-II^hi^CD135^+^CD115^−^ DC cells increased significantly in the state of sepsis [113]. Mechanistically, it has been implicated that ERS can activate NLRP3 inflammasome [114, 115]. Our data confirmed that ERS was overactivated during sepsis, which facilitated ERS-associated NLRP3 activation. Furthermore, the study discovered that Sestrin2 (SESN2) effectively suppressed excessive activation of the NLRP3 inflammasome and the resulting caspase-1-dependent pyroptosis, leading to an enhanced prognosis in sepsis. This was achieved by stabilizing the endoplasmic reticulum (ER), highlighting the importance of identifying novel therapeutic targets for sepsis treatment [113].

**Fig. 4:**
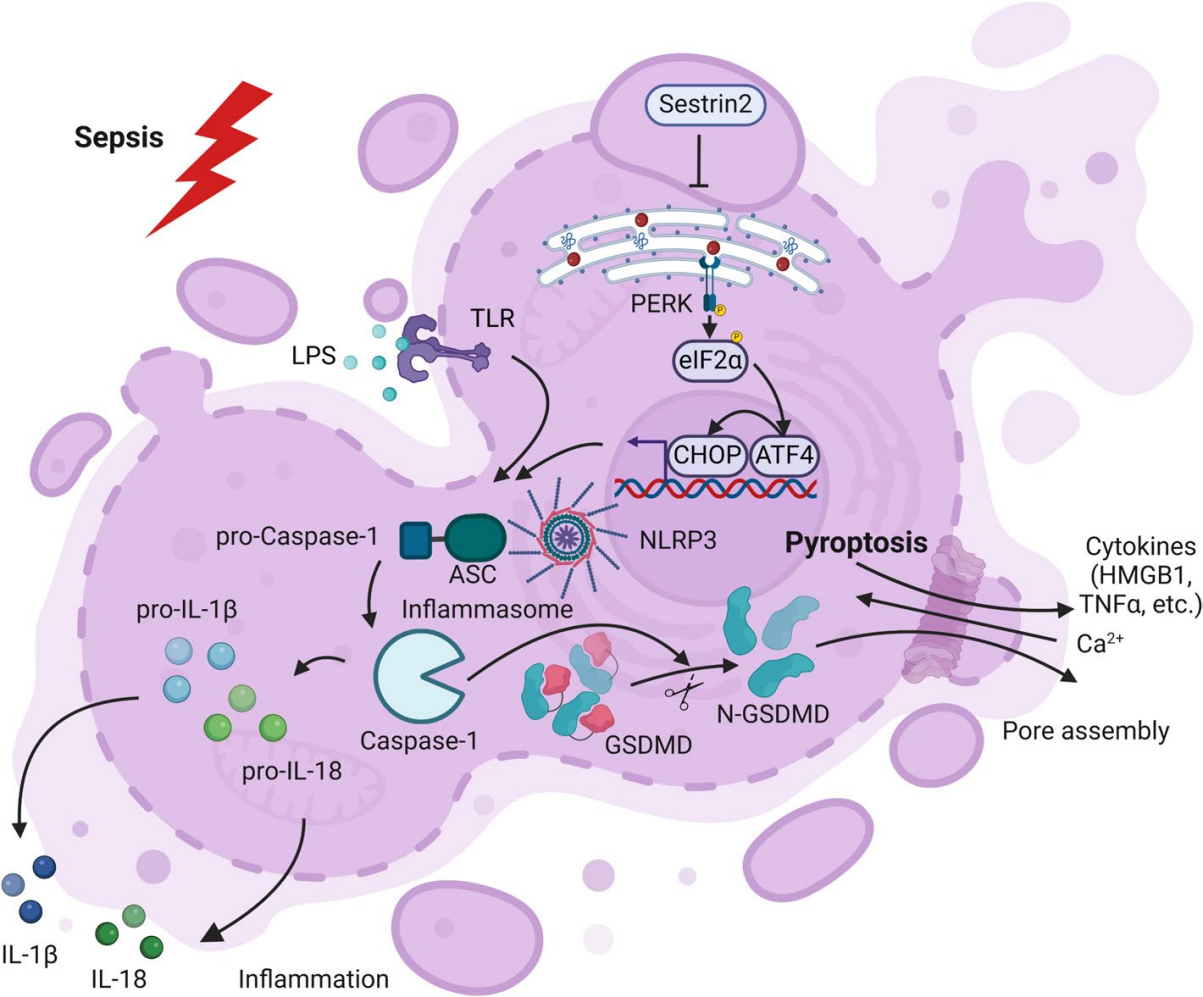
Pyroptosis of dendritic cells (DCs) in sepsis. Pyroptosis of DCs is mainly recognized by TLRs, then activates inflammasomes, including NLRP3 and other inflammasomes. Pro-caspase-1 in inflammasomes activates the active form caspase-1, which cleaves Gasdermin family proteins such as GSDMD. Its N-terminal oligomerizes and punches pores on the cell membrane, resulting in cell membrane rupture. In addition, caspase-1 cleaves IL-1β and IL-18 precursors, so they become active and are secreted outside the cell through pores. Recent studies have demonstrated that excessive ERS in sepsis will lead to inflammasome activation and promote pyroptosis of DCs. Sestrin2 can alleviate the above harmful process, inhibit sepsis-induced pyroptosis of DCs, and reduce the mortality of septic mice. LPS, lipopolysaccharide; TLR, Toll-like receptor; PERK, PKR-like endoplasmic reticulum kinase; ATF4, activating transcription factor 4; eIF2α, eukaryotic initiating factor 2α; CHOP, C/EBP homologous protein; NLRP3, nucleotide binding oligomerization domain (NOD)-like receptor protein 3; ASC, apoptosis speck-like protein containing a caspase recruitment domain; GSDMD, Gasdermin D; IL, interleukin; TNF, tumor necrosis factor; HMGB1, high mobility group box-1 protein

In the pathogenesis of sepsis, DCs also exhibit harmful effects on organisms through the nonclassical pathway, known as caspase11-dependent pyroptosis. Zanoni et al. found that LPS stimulation induced caspase-11-dependent pyroptosis of DCs with IL-1 release [116]. Kumari et al. further demonstrated the harmful impact of caspase-11 on CD11c^+^ cells in LPS-induced septic shock [117] (Fig. 4).

### Ferroptosis

Ferroptosis, a distinct form of cell death introduced in 2012, is induced by the oxidation of phospholipids dependent on iron [105]. Although no chromatin condensation or loss of plasma membrane integrity is found in the morphological characteristics, mitochondrial concentration, reduction in mitochondrial cristae, and increase of membrane density can be observed. Different cellular metabolic pathways, such as redox homeostasis, iron metabolism, mitochondrial function, and the metabolism of amino acids, lipids, and sugars, along with various disease-related signaling pathways, regulate ferroptosis [118]. Ferroptosis can be induced by various substances, including erastin and RSL3 as experimental reagents, sorafenib, sulfasalazine, statins, and artemisinin as approved drugs, ionizing radiation, and cytokines such as IFN-γ and TGF-β1 [119]. The regulatory process of ferroptosis involves both the conventional pathway mediated by glutathione peroxidase 4 (GPX4) and the alternative pathway that is independent of GPX4 [120]. Cystine is transported into the cell through the reverse transporter of cystine/glutamate (system Xc^−^) in the canonical controlling pathway of GPX4. Subsequently, it undergoes reduction to cysteine in a manner that depends on glutathione (GSH) and/or thioredoxin reductase 1 (TXNRD1). These processes promote the biosynthesis of GSH. GSH functions as a powerful suppressor that enhances the intracellular conversion of phospholipid hydroperoxides (PLOOHs) to PLOOHs-corresponding alcohols (PLOHs) by acting as a coenzyme of GPX4. PLOOHs, known as lipid-derived reactive oxygen species (ROS), are believed to function as the primary agents responsible for executing ferroptosis. The most extensively researched noncanonical regulatory pathways of ferroptosis are the ferroptosis suppressor protein 1 (FSP1)/ubiquinone (CoQ10) system and the GTP cyclohydrolase 1 (GCH1)-tetrahydrobiopterin (BH4) system [118]. Remarkably, ferritin degradation (referred to as ferritinophagy), a burgeoning area of interest in ferroptosis investigation, is responsible for the elevation of Fe^2+^ levels within cells. The aggregation of Fe^2+^ produces hydroxyl radicals (·OH) through the Fenton reaction and induces lipid peroxidation and ferroptosis [119].

**Fig. 5:**
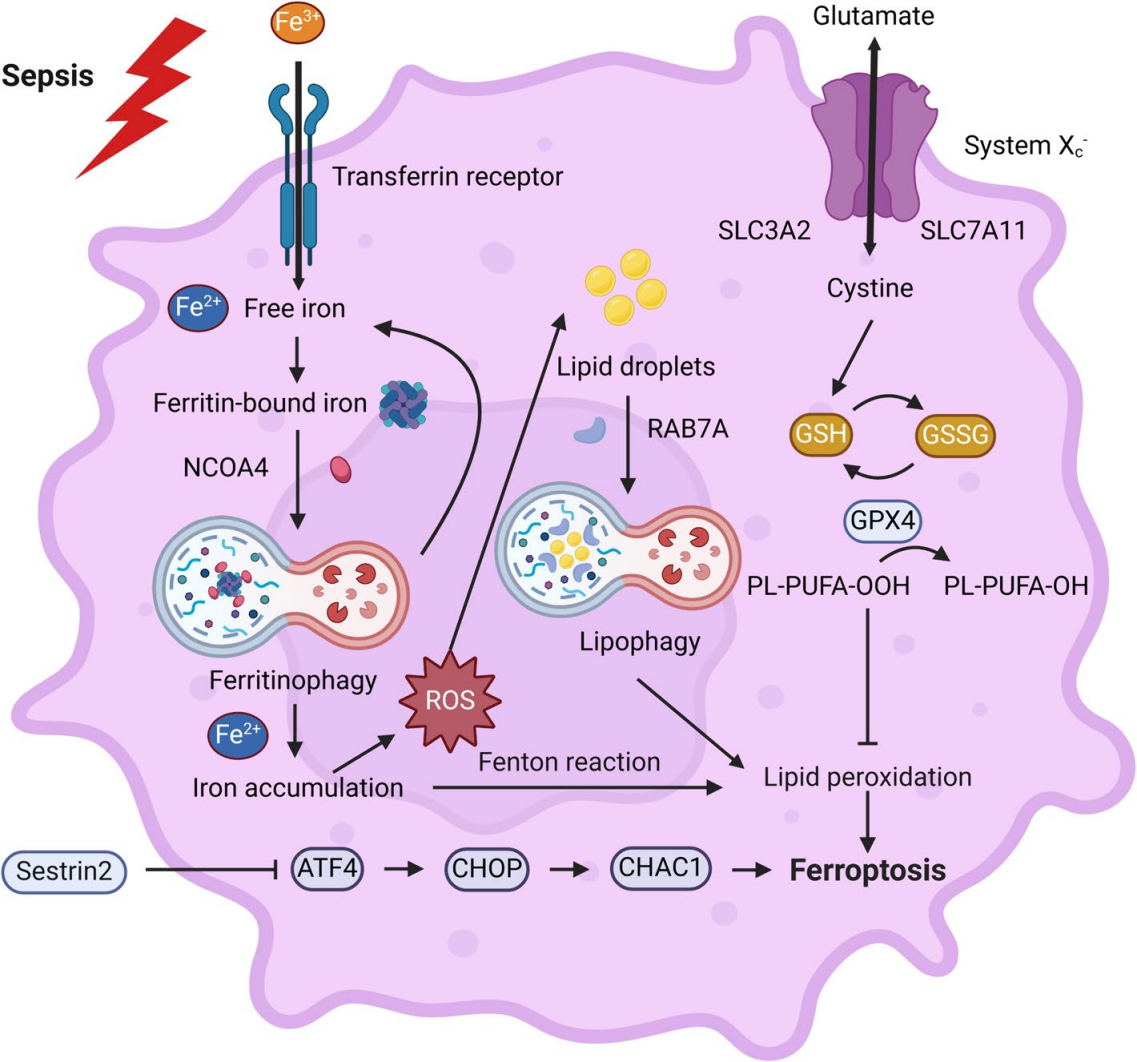
Ferroptosis of dendritic cells (DCs) in sepsis. The primary mechanism of ferroptosis is the accumulation of ferrous iron in the intracellular and the initiation of lipid peroxidation through the Fenton reaction. GPX4 is the only glutathione peroxidase (GPX) used for lipid peroxide reduction in cells. Inactivation of GPX4 will contribute to lipid peroxidation and then induce ferroptosis. Many studies have indicated that DCs in a septic state have obvious ferroptosis. Sestrin2 can protect DCs against sepsis-induced ferroptosis through the ATF4–CHOP–CHAC1 signaling pathway. GPX4, glutathione peroxidase 4; GSH, glutathione; PLOOH, phospholipid hydroperoxide; PLOH, PLOOHs-corresponding alcohol; ROS, reactive oxygen species; ATF4, activating transcription factor 4; CHOP, C/EBP homologous protein; CHAC1, cation transport regulator homolog 1

Increasing evidence has been suggested that ferroptosis appears to be vital in the development of sepsis [103, 121, 122]. Many reports in animal models of sepsis have indicated that ferroptosis is increased and closely related to sepsis-induced cardiac, liver, and lung injury secondary to LPS-induced endotoxemia and CLP surgery [123–126]. Ferroptosis inhibitors such as ferrostatin-1 (Fer-1), sevoflurane (Sev), panaxydol (PX), and irisin can abate sepsis-induced multiple organ dysfunction and improve survival rates [124–129]. Clinical trials demonstrated a reduction in serum irisin levels among septic individuals, which exhibited a negative correlation with the acute physiology and chronic health evaluation (APACHE) II score. Treatment with irisin may offer therapeutic potential in managing sepsis [126]. Similarly, changes in the regulation of iron levels in the body were noted in individuals with sepsis, and increased levels of iron in the blood and ferritin were found to have a positive association with the sequential organ failure assessment (SOFA) score and patient mortality in sepsis cases [130]. In our study, we noticed that DCs in a septic state presented with ferroptosis, which could significantly hinder DC maturation. To this end, the administration of Fer-1 could relieve such impact. Further research demonstrated that SESN2 protected DCs against sepsis-induced ferroptosis through an activating transcription factor 4 (ATF4)–C/EBP homologous protein (CHOP)–cation transport regulator homolog 1 (CHAC1)-dependent manner [50, 131] (Fig. 5).

### Autophagy-dependent cell death

**Fig. 6:**
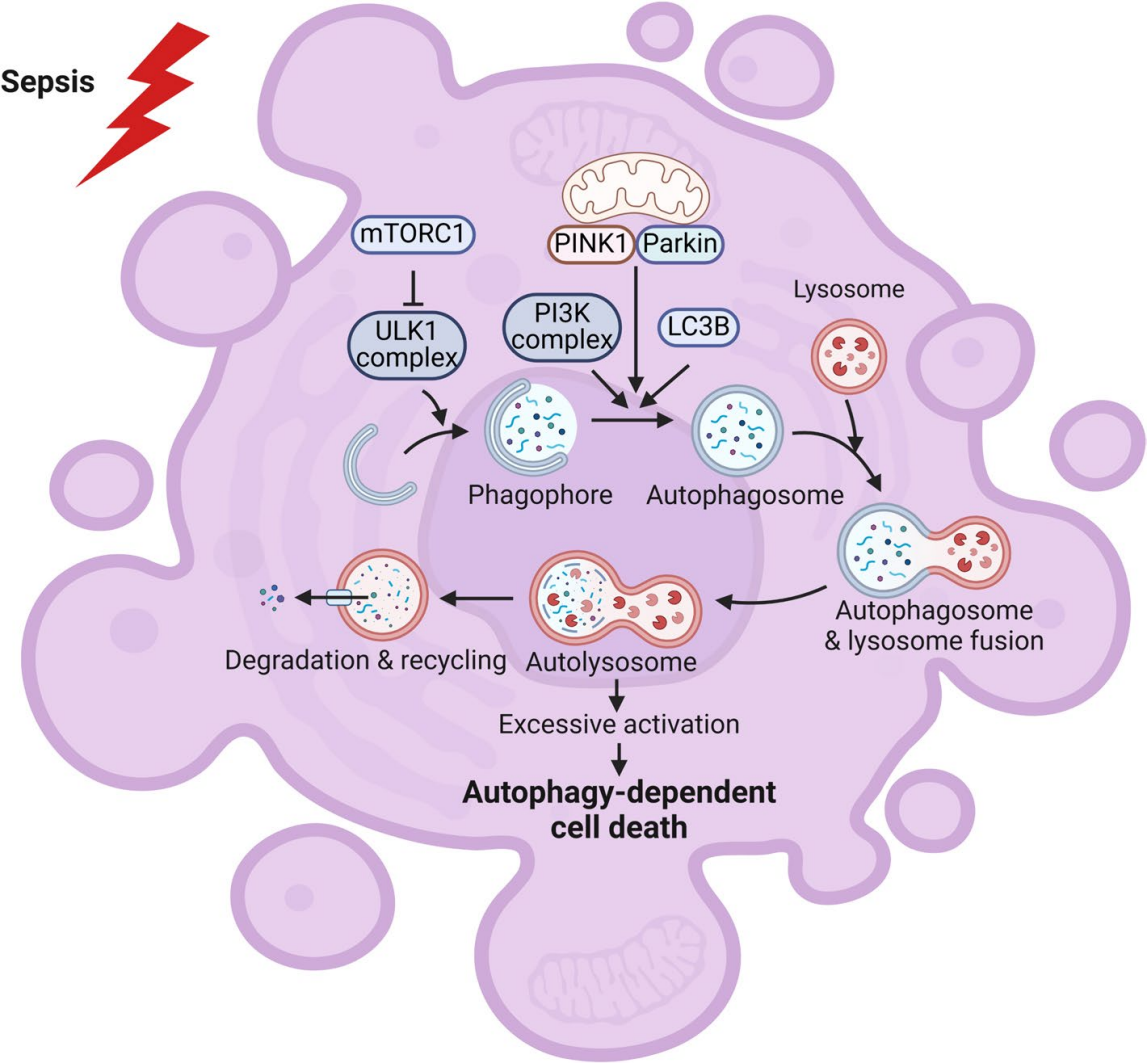
Autophagy-dependent cell death (ADCD) of dendritic cells (DCs) in sepsis. Autophagy refers to the process of autophagosome formation, isolation of cytosol and organelles, and transport to lysosomes for degradation and recycling of macromolecules. However, when autophagy is overactivated, it can lead to ADCD. mTORC1, mammalian target of rapamycin C1; ULK1, unc-51-like kinase 1; PI3K, phosphoinositide 3 kinase; PINK1, PTEN-induced putative kinase 1; LC3B, microtubule-associated protein 1 light chain 3 β

ADCD is a type of RCD that relies on the autophagic machinery (or its components) mechanistically [132]. The traditional comprehension of ADCD is a death due to excessive self-consumption of organelles and cytoplasmic content that depends on autophagy genes and requires autophagy flux [133, 134]. Recently, this has been questioned due to the identification of autosis as a form of ADCD, which is a death due to activation of the Na^+^/K^+^-ATPase pump, changes in membrane osmolarity, and ion transport, which is dependent on autophagy genes but not autophagy flux [135, 136]. Currently, there are three ADCD types: excessive autophagy, excessive organelle-specific autophagy, and autosis [137, 138]. Autophagy can protect cells or trigger cell death [9, 139]. Autophagy was shown to be involved in DC functions at several levels [57, 140, 141]. A loss of autophagy in DCs caused a sepsis-like condition, including tissue inflammation and hyperproduction of inflammasome-related cytokines [142]. Impaired PINK1/Parkin-mediated mitophagy renders apoptosis of DCs, resulting in sepsis-induced immunosuppression [143]. Due to the extensive intersection of autophagy with apoptotic and necrotic signals and the complexity of the association between ADCD and apoptosis, necrosis, and ferroptosis, the definition of ADCD has been controversial [144–148]. Exploring the precise regulation of “lethal” and “nonlethal” autophagy flux in DCs during sepsis may provide a new therapeutic approach for sepsis [149] (Fig. 6).

## Targeting DCs during sepsis

Since sepsis-induced immunosuppression mediated by DC dysfunction is crucial for the prognosis of septic patients. In the past decades, there have been a number of immune-modulatory therapies that could impact DC function and survival, although they are not DC specific, including anti-PD-1, anti-PDL1, anti-FAS, anti-CTLA4, etc. [5, 8, 26, 150, 151]. Some of the clinical trials related to it are still ongoing. (ClinicalTrials.gov ID NCT01161745 and ClinicalTrials.gov ID NCT05126537). Targeting DC dissonance brings about the possibility of the effective treatment of sepsis. Current measurements of DCs can be mainly divided into two categories: modifying immune functions of DCs and inhibiting cell death of DCs (Table 1).

**Table 1:** Immunotherapy targeting DCs in sepsis

Strategies	Treatment	Major functions	References
Modifying functions of DCs	Glucocorticoids	Reducing the production of IL-12 and augmenting the loss of CD8^+^ DCs	[152–160]
	Thymosin α1	Enhancing the expression of DCs surface molecules; upregulating the expression of TLR2 and TLR9 on the surface of DCs; promoting the secretion of IL-2, IL-12, and IFN-α	[161–165]
	TLR4 antagonist FP7 and Eritoran	Inhibiting cytokines storm and glycolysis reprogramming of DCs	[59, 166–171]
	TLR2-derived peptides	Upregulating CD14 activity and promoting antigen-mediated DCs maturation; increasing the release of IL-12 and IFN-γ and decreasing the release of TNF-β; promoting the differentiation of T cells toward Th1	[172, 173]
	PLA2	Increasing the expression of surface molecules of DCs and promoting DC maturation	[174, 175]
	Anti-HMGB1 antibody	Augmenting maturation of DCs and T cell polarization toward Th1	[176–180]
	Anti-C5a antibody	Enhancing IL-12^+^ DCs in the abdominal cavity and inhibiting them in peripheral blood and lymph nodes	[181–191]
Inhibiting cell death of DCs	Silencing of miR-142-p, miR155, and miR-146a/b	Inhibiting the apoptosis of DCs; increasing production of proinflammatory cytokines such as IL-12p70, IL-6, TNF-α, and IFN-γ	[192–194]
	Overexpressed VIM	Inhibiting apoptosis of DCs Inhibiting production of proinflammatory cytokines such as IL-2, IL-10, and IFN-α	[195]
	Dexmedetomidine	Inhibiting the apoptosis of DCs; downregulating the production of sepsis-induced inflammatory mediators, including TNF-α and IL-6	[196–198]

DCs, dendritic cells; IL, interleukin; IFN, interferon; TNF, tumor necrosis factor; Treg, regulatory T cells; TLR, Toll-like receptor; HMGB1, high mobility group box-1 protein; PLA2, phospholipase A2; miRNAs, microRNAs; VIM, vimentin

### Modifying immune functions of DCs

The cytokine IL-12 facilitates the release of inflammatory substances, leading to the secretion of IFN-γ and causing lethality in septic shock induced by LPS [152]. Glucocorticoids (GCs) are vital regulatory compounds in the body that have a significant impact on the body’s development, growth, metabolism, and immune function. They also have potent anti-inflammatory and immunosuppressive properties [153–155]. It has been shown that endogenous GCs inhibit the response of DCs to LPS exposure, reduce the production of IL-12, and augment the loss of CD8^+^ DCs, thereby playing a lifeguard role in the high-inflammatory phase of sepsis [152, 156]. According to the present guidelines for sepsis treatment, it is advised to administer intravenous corticosteroids to adult patients experiencing septic shock and requiring continuous vasopressor support [157]. In individuals suffering from severe sepsis but not experiencing septic shock, the administration of hydrocortisone did not result in a decrease in the likelihood of developing septic shock within a 14-day period when compared with the use of placebo controls (ClinicalTrials.gov ID NCT00670254) [158]. Nevertheless, the precise dosage, initiation timing, and duration of corticosteroids are still unclear. The hydrocortisone-plus fludrocortisone group had a lower mortality rate compared with the placebo group in clinical trials involving adults with septic shock (ClinicalTrials.gov ID NCT00625209) [159]. Clinical trials of the effects of early use of dexamethasone in patients with high-risk sepsis are ongoing (ClinicalTrials.gov ID NCT05136560) [160]. The safety and efficacy of glucocorticoids in treating sepsis and the concrete signaling pathways need to be further clarified.

Thymosin α1 is a small-molecule polypeptide purified from the calf thymus with a nonspecific immune effect. Its chemical and spatial structure are apparent; its main active ingredient consists of 28 amino acids. Research has indicated that the administration of thymosin α1 can improve the capacity of T cells to generate and release IFN-γ, as well as increase the HLA-DR expression in monocytes [161]. Likewise, it has the potential to enhance the presentation of surface markers on DCs, such as CD40, CD80, MHC-I, and MHC-II, thereby stimulating the differentiation and activation of DCs [162]. Furthermore, thymosin α1 has the ability to increase the levels of TLR2 and TLR9 on the outer layer of DCs [163], as well as enhance the release of inflammatory cytokines such as IL-2, IL-12, and IFN-α [164]. Preclinical studies have documented that it can help restore immune response and improve the survival of patients with sepsis [165]. Clinical trials on the long-term prognosis of immunotherapy with thymosin α1 in septic patients are ongoing (ClinicalTrials.gov ID NCT04901104). Studying the regulation of the TLRs signaling pathway is significant because of abnormal TLR involvement in sepsis pathogenesis. It has been established that TLR2 and TLR4 are critically involved in the sepsis-induced depletion of splenic DCs [59, 166, 167]. Further studies showed that the TLR4 antagonist FP7 inhibited LPS-induced cytokine production and DC glycolysis reprogramming, and protected mice from fatal viral sepsis, most likely by reducing the TLR4-dependent cytokine storm mediated by DAMPs such as HMGB1 [168]. Positive results were observed in both phase I and II trials investigating the impact of the MD2-TLR4 antagonist Eritoran on poor outcomes in patients with severe sepsis. However, phase III trials demonstrated that Eritoran did not decrease the 28-day mortality in patients with severe sepsis when compared with placebo controls (ClinicalTrials.gov ID NCT00334828) [169]. CD14, as a co-receptor of TLR7 and TLR9, plays a role in recognizing the common signals of pathogen-associated molecular patterns (PAMPs) and can be a potential target for regulating DC-mediated Th1 differentiation [170, 171]. TLR2-derived peptides can augment CD14 activity and promote antigen-induced DC maturation by upregulating MHC-II, CD80, and CD86 expressions. In this regard, the peptide increases the release of IL-12 and IFN-γ from DCs, inhibits the formation of TNF-β, promotes the differentiation of T cells toward Th1, and improves the immunosuppressive state [172]. Other studies have implicated that immunosuppression induced by sepsis is related to serum TLR-9 level [173]. Taken together, targeting TLRs may be an exciting and promising area of sepsis therapy.

Phospholipase A2 (PLA2) is an enzyme that facilitates the hydrolysis of the 2-acyl moiety on glycerol molecules of phospholipids. In septic patients, there is a notable increase in the serum levels of PLA2, particularly the secretory PLA2-IIA (sPLA2-IIA), which can serve as a dependable indicator for diagnosing sepsis (ClinicalTrials.gov ID NCT03953404) [174]. sPLA2 can enhance the expression of CD86, CD80, CD83, and CD40 on the surface of DCs, promote DC maturation, and improve the prognosis of sepsis (ClinicalTrials.gov ID NCT00034476) [175].

HMGB1 is a potent proinflammatory cytokine at the late stage of sepsis and is associated with delayed death from endotoxin and sepsis [176]. HMGB1 plays a dual role in regulating the immune functions of DCs. It activates DC maturation and T-cell polarization toward Th1 at a specific concentration and stimulation time. Nevertheless, an overabundance of HMGB1 stimulation can result in atypical development and impaired immune function of DCs [177]. Anti-HMGB1 antibody treatment and specific inhibition of DC secretion of HMGB1 by small interfering RNA (siRNA) of HMGB1 significantly reduce sepsis-induced mortality, which may provide a treatment strategy for sepsis [178, 179]. HMGB1 is a marker of cell damage and activation and is known to increase in ICU patients. It was found in clinical trials that HMGB1 levels elevated in study participants hospitalized 3–6 months after ICU admission, although there was no association with the primary outcome, physical performance (ClinicalTrials.gov ID NCT02914756) [180]. Another clinical study with regard to the pro-inflammatory effects of blood platelets (including plasma concentration in HMGB1) in critically ill patients with septic shock is ongoing (ClinicalTrials.gov ID NCT04080453).

Basically, C5a is a necessary complement and a powerful chemokine overactivated during sepsis [181, 182]. It modulates the balance of cytokines and DC distribution by regulating the expression of adherent cytokines [183–186]. C5a promotes the movement of IL-12^+^ DCs from the peritoneal cavity to both lymph nodes and peripheral blood. Additional research indicates that IL-12^+^ DCs facilitate the proliferation of pathogenic IL-17^+^ T helper cells (Th17) and IFN-γ^+^ T helper cells (Th1) [181, 187, 188]. Moreover, excessive expression of IL-12^+^ DCs detrimentally affects the host during a septic condition [189, 190]. In septic mice treated with anti-C5a antibody, IL-12^+^ DCs in peripheral blood and lymph nodes decreased, while IL-12^+^ DCs in the abdominal cavity increased and exerted a protective impact, thereby improving the prognosis of mice subjected to septic challenge [181]. In a clinical study, a new extracorporeal treatment for sepsis showed promising results. By using immunoadsorption (IA) therapy, the levels of circulating endotoxin, IL-6, and C5a were significantly reduced, leading to the reversal of antigen-presenting cell deactivation and improvement in organ functions (ClinicalTrials.gov ID NCT00146432) [191].

### Inhibiting cell death of DCs

Currently, the apoptosis of DCs represents the most extensively studied attempt to restore the number of DCs.

MicroRNAs (miRNAs) are a type of RNA that is not involved in coding and is produced by genes within the organism. They are approximately 22 nucleotides long and play a role in controlling gene expression after transcription. Many studies have demonstrated that a variety of miRNAs are induced to express during the development, maturation, and differentiation of monocytes into DCs, including miR-142-p, miR-155, and miR-146a/b, which will lead to increased apoptosis of DCs and change the function of DC-mediated cytokines. Inhibiting these genes greatly reduces the apoptosis of DCs and enhances the synthesis of proinflammatory cytokines such as IL-12p70, IL-6, TNF-α, and IFN-γ, thereby substantially enhancing the survival rates in response to endotoxin-induced conditions [192–194]. Studies have been carried out to investigate the possible function of miRNAs (miR-223, miR-15a, miR-16) in controlling the growth and death of lymphocytes during sepsis (ClinicalTrials.gov ID NCT02756559). These findings on miRNAs suggest that miRNAs can be used as a new strategy for treating sepsis.

The findings of the clinical trial indicated that individuals diagnosed with sepsis and septic shock exhibited notably elevated serum vimentin (VIM) levels in comparison with the control group. In cell experiments, it was observed that the upregulation of caspase-3 expression was significant in VIM-deficient cells when compared with control cells. In contrast, caspase-3 was reduced by nearly 40% in cells that overexpressed VIM. IL-2, IL-10, and IFN-α levels were significantly lower in VIM-deficient cells than those in control cells, while there was no significant change in cells with high VIM expression. These findings indicate that VIM regulates apoptosis and inflammatory response of lymphocytes. The identification and prediction of patients’ outcomes with sepsis or septic shock could potentially benefit from focusing on VIM as a new approach [195] (ClinicalTrials.gov ID NCT03253146).

Multiple clinical trials have demonstrated that sedation strategies using dexmedetomidine mitigate excessive inflammation, improve renal function, shorten the time required for mechanical ventilation, and reduce mortality in patients with sepsis. The potential causes could be associated with dexmedetomidine in reducing the generation of sepsis-triggered inflammatory substances, such as TNF-α and IL-6, and preventing apoptosis [196–198] (ClinicalTrials.gov ID NCT01760967).

## Future perspectives and remarks

Septic shock and the consequent MODS, which are leading causes of death in critically ill patients, often arise as a result of sepsis, a frequent complication in individuals with trauma/burns, infection, and severe internal/surgical conditions. The immune response of the host during sepsis encompasses intricate pathophysiological mechanisms. The exact molecular mechanism and key regulatory pathways of immune dysfunction in sepsis remain to be elucidated [7, 9, 10, 199]. In clinical practice, it is imperative to monitor and control the immune function of patients suffering from sepsis [200]. Understanding how immune cells change in function and number in sepsis can aid in elucidating its pathophysiology and improving prognosis. DCs are known to be the most powerful APCs in the body and essential regulatory cells of the immune system, and they can be roughly divided into three subgroups: cDCs, pDCs, and FDCs. In the pathogenesis of sepsis, the functions and quantity of DCs undergo significant changes; the main symptom is an impairment of DCs and a substantial decrease in their amount. The dissonance of DCs is characterized by abnormal surface molecular expression, cytokine secretion, and dampened T cell-stimulatory capacity. Marked reduction in the number of DCs during sepsis involves various pathways, with apoptosis being the most extensively investigated. Lately, there has been a growing interest in research concerning recently identified RCD, which encompasses necroptosis, pyroptosis, ferroptosis, and ADCD. Modulating the RCD of DCs in sepsis would be a new treatment target. Targeting DCs to regulate host immunity has become a crucial research field in sepsis due to their critical role in the immune response. In recent times, numerous approaches have been formulated and effectively employed to mitigate atypical immune reactions during the advancement of sepsis, encompassing the alteration of DCs’ functionalities and the suppression of DCs’ cell death. Although these treatments are still in preclinical trials and have not proven effective for septic patients, they hold immense potential for clinical management.

To conclude, further study is needed to understand the potential role of DCs in sepsis. A thorough investigation into the molecular basis of DCs during sepsis and the development of novel treatment strategies targeting DCs might improve immune-modulatory processes against septic insults.

## Data Availability

Not applicable.

## Abbreviations

ICU: Intensive care unit
LMICs: Low- and middle-income countries
DCs: Dendritic cells
APCs: Antigen-presenting cells
TCRs: T cell surface receptors
MHC: Major histocompatibility complex
HLA-DR: Human leukocyte antigen-DR
MHC-II: MHC class II
IL: Interleukin
scRNA-seq: Single-cell RNA sequencing
CytoF: Cytometry by time-of-flight
PCD: Programmed cell death
ADCD: Autophagy-dependent cell death
cDCs: Classical or conventional DCs
pDCs: Plasmacytoid dendritic cells
FDCs: Follicular dendritic cells
CMPs: Common myeloid progenitors
LCs: Langerhans cells
TLRs: Toll-like receptors
CLPs: Common lymphoid progenitor
IFN: Interferon
CXCL: CXC chemokine ligand
NF-κB: Nuclear factor κ-B
MPCs: Mesenchymal progenitor cells
mregDCs: Mature DCs enriched in immunoregulatory molecules
CLP: Cecal ligation and puncture
BALF: Bronchoalveolar lavage fluid
TNF: Tumor necrosis factor
TGF: Transforming growth factor
Tregs: Regulatory T cells
HSPCs: Hematopoietic stem cells and hematopoietic progenitor cells
ERS: Endoplasmic reticulum stress
TNFAIP8L2: Tumor necrosis factor α-induced protein 8 like-1
PD-L1: Programmed cell death-ligand 1
PD-1: Programmed death 1
TAK1: TGF-β-activated kinase 1
JNK: C-Jun N-terminal kinases
PTEN: Protein tyrosine phosphatase
PINK1: PTEN-induced putative kinase 1
NCCD: Nomenclature Committee on Cell Death
ACD: Accidental cell death
RCD: Regulated cell death
APAF-1: Apoptotic protease activating factor-1
TNFR: TNF receptor
Fas: Factor associated suicide
TRAIL: TNF-related apoptosis-inducing ligand
TRAILR: TRAIL receptor
Bcl-2: B-cell lymphoma-2
PI3K: Phosphoinositide 3 kinase
LPS: Lipopolysaccharide
A-SMase: Acid sphingomyelinase
PKG: CGMP-dependent protein kinases
iNOS: Inducible nitric oxide synthase
IRF: Interferon regulatory factor
MyD88: Myeloid differentiation primary response gene 88
NFAT: Calcineurin-activated T nuclear factor
RIPK: Receptor-interacting protein kinase
MLKL: Mixed-lineage kinase domain-like protein
DAMPs: Damage-related molecular patterns
mtDNA: Mitochondrial DNA
HMGB1: High mobility group box-1 protein
LDH: Lactate dehydrogenase
MODS: Multiple organ dysfunction syndrome
ATF6: Activating transcription factor 6
MDDCs: Monocyte-derived DCs
rh-APC: Recombinant human-activated protein C
PRR: Pattern recognition receptor
NLR: NOD-like receptor
GSDMD: Gasdermin D
SESN2: Sestrin2
GPX4: Glutathione peroxidase 4
GSH: Glutathione
TXNRD1: Thioredoxin reductase 1
PLOOHs: Phospholipid hydroperoxides
PLOHs: PLOOHs-corresponding alcohols
ROS: Reactive oxygen species
FSP1: Ferroptosis suppressor protein 1
CoQ10: Ubiquinone
GCH1: GTP cyclohydrolase 1
BH4: Tetrahydrobiopterin
Fer-1: Ferrostatin-1
Sev: Sevoflurane
PX: Panaxydol
APACHE: Acute physiology and chronic health evaluation
SOFA: Sequential organ failure assessment
ATF4: Activating transcription factor 4
CHOP: C/EBP homologous protein
CHAC1: Cation transport regulator homolog 1
GCs: Glucocorticoids
PAMPs: Pathogen-associated molecular patterns
PLA2: Phospholipase A2
sPLA2-IIA: Group IIA secretory PLA2
siRNA: Small interfering RNA
Th: T helper cells
miRNAs: microRNAs
VIM: Vimentin
IA: Immunoadsorption
